# A case report of transcatheter aortic valve replacement in a patient with Sjögren's syndrome and aortic stenosis

**DOI:** 10.1093/ehjcr/ytad622

**Published:** 2023-12-11

**Authors:** Zhenyu Yang, Wei Fang, Qiuhe Wang, Yan Li

**Affiliations:** Department of Cardiology, Tangdu Hospital, Air Force Medical University, Tangdu Hospital, 1 Xinsi Road, Baqiao District, Xi'an, Shaanxi, Province, China; Department of Cardiology, Tangdu Hospital, Air Force Medical University, Tangdu Hospital, 1 Xinsi Road, Baqiao District, Xi'an, Shaanxi, Province, China; Department of Cardiology, Tangdu Hospital, Air Force Medical University, Tangdu Hospital, 1 Xinsi Road, Baqiao District, Xi'an, Shaanxi, Province, China; Department of Cardiology, Tangdu Hospital, Air Force Medical University, Tangdu Hospital, 1 Xinsi Road, Baqiao District, Xi'an, Shaanxi, Province, China

**Keywords:** Sjögren's syndrome, aortic stenosis, transcatheter aortic valve replacement, thrombocytopaenia, case report

## Abstract

**Background:**

The co-existence of Sjögren's syndrome and aortic stenosis (AS) is infrequent, and there lack cases of transcatheter aortic valve replacement (TAVR) for these patients with detailed management decision-making.

**Case summary:**

We report a case of a female patient who had AS and Sjögren's syndrome with leukopaenia and thrombocytopaenia. To overcome co-existing hyper-coagulation and high thrombogenesis risk, difficult lifetime valve management, and high infection risk, we performed TAVR with 3D printing and formulated antithrombotic and antibiotic schemes.

**Conclusion:**

This case provided a successful experience of TAVR in patients with Sjögren's syndrome. Long-term follow-up will be conducted, and optimization of the therapeutic regimen requires further exploration.

**Learning points**

- Sjögren's syndrome may damage the structure of valve through autoimmune reactions.
- Attention should be paid to the effects of inflammation and coagulation function in valve management.

## Introduction

Sjögren's syndrome is a less-understood autoimmune disorder with an insidious onset and variable symptoms. The core symptoms are xerophthalmia and xerostomia with multi-organ dysfunction, but rarely involving the heart valves. A previous study showed that such valvular damage was usually asymptomatic and the predominant type was regurgitation.^1^ One possible explanation could be that the Sjögren's syndrome antibody (anti-Ro) causes aortic valve damage via a wide range of inflammatory reactions.^2^ Sjögren's syndrome is associated with thrombocytopaenia and leukopaenia, increasing the risk of bleeding and infection.^3^ We hereby present a rare case of Sjögren's syndrome with severe aortic stenosis (AS) and thrombocytopaenia.

## Summary figure

**Table:** 

Timeline	
08/01/2023	Re-visited for repeated joint pains, keratoconjunctivitis sicca, and xerostomia
09/01/2023	Echocardiography screening tests indicated severe aortic stenosis (AS)
07/02/2023	Transferred to Cardiology Department
08/02/2023	Echocardiography: severe AS with an effective orifice area of 0.68 cm^2^ accompanied with aortic valve calcification
09/02/2023	Diagnosis of severe AS and Sjögren's syndrome confirmed
11/02/2023	Computed tomography angiography (CTA): a bicuspid aortic valve of type 0 and the valve calcification area was 248 mm^3^ (HU 850)
14/02/2023	Performed transcatheter aortic valve replacement and cefazolin sodium was given
16/02/2023	The patient’s platelet decreased to 30 × 10^9^/L. Cefazolin sodium was suspended, and low-dose immunoglobulin was infused to rapidly rise platelet counts.
20/02/2023	Echocardiography: haemodynamic parameters were improved
21/02/2023	Discharge
31/03/2023	CTA: hypo-attenuated leaflet thickening. Oral warfarin was given to replace clopidogrel

## Case presentation

A 49-year-old female patient was admitted to our hospital complaining of xerophthalmia and xerostomia, accompanied by obvious chest tightness, shortness of breath, and palpitations. She was diagnosed with connective tissue disease in 2016 and had discontinued methylprednisolone 8 mg/day a month ago. She suffered a cerebral infarction 2 years ago and had a 6-year history of hypertension. On physical examination, a grade 4/6 systolic murmur was heard in the aortic and tricuspid valve auscultation area. Echocardiography screening test results indicated severe AS and mild tricuspid regurgitation (TR). At the suggestion of cardiologists, sacubitril/valsartan 100 mg/day was given to control blood pressure and improve ventricular remodelling, and methylprednisolone 28 mg/day was used for anti-inflammation, with plans for perform an aortic valve replacement.

**Figure 1:**
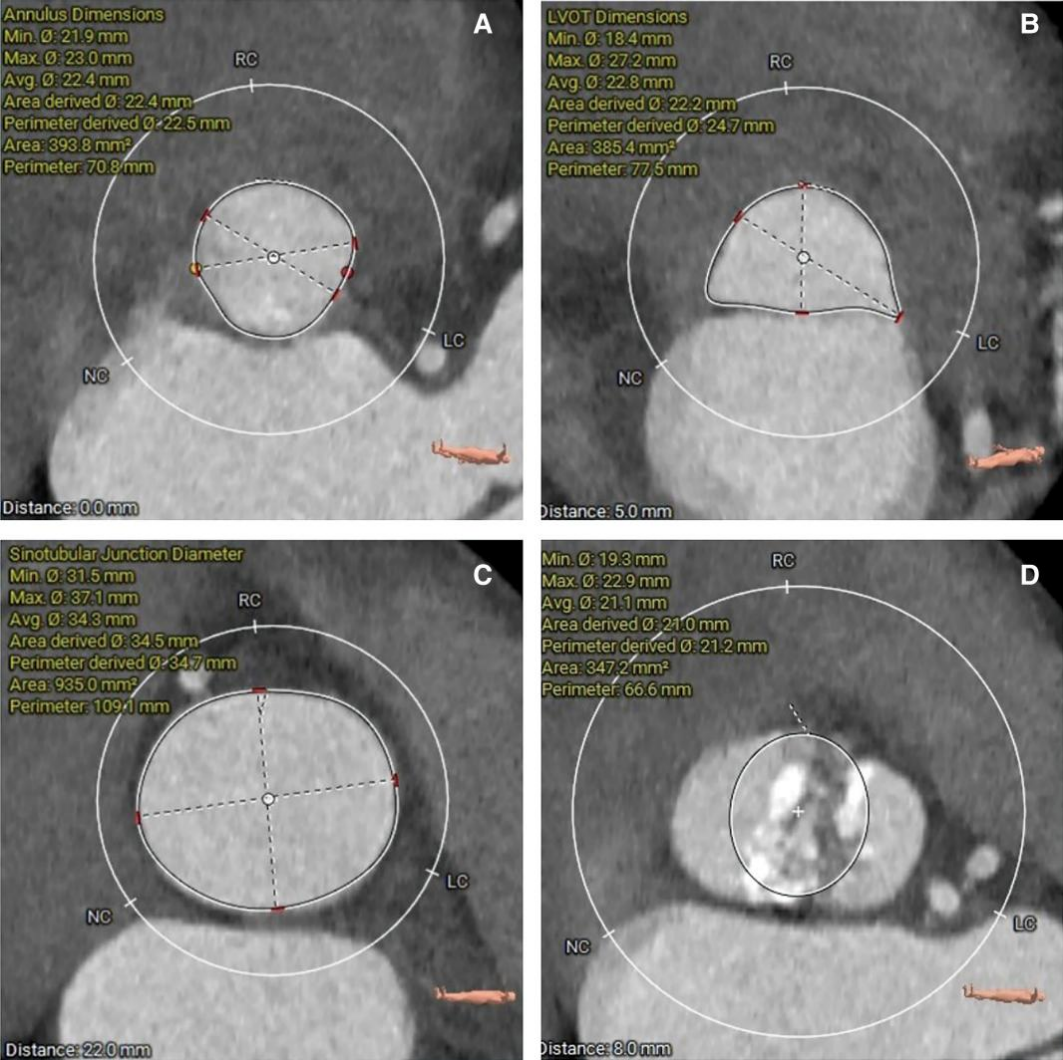
Aortic computer tomography before transcatheter aortic valve replacement. Calcification is mainly distributed at the bottom of the right coronary cusp and non-coronary cusp, extending into the middle of the valve leaflet, left coronary sinus leaflet, and at the confluence of the two sinuses. (*A*) Annulus; (*B*) 5 mm below the annulus; (*C*) sinotubular junction; (*D*) 8 mm above the annulus.

**Figure 2:**
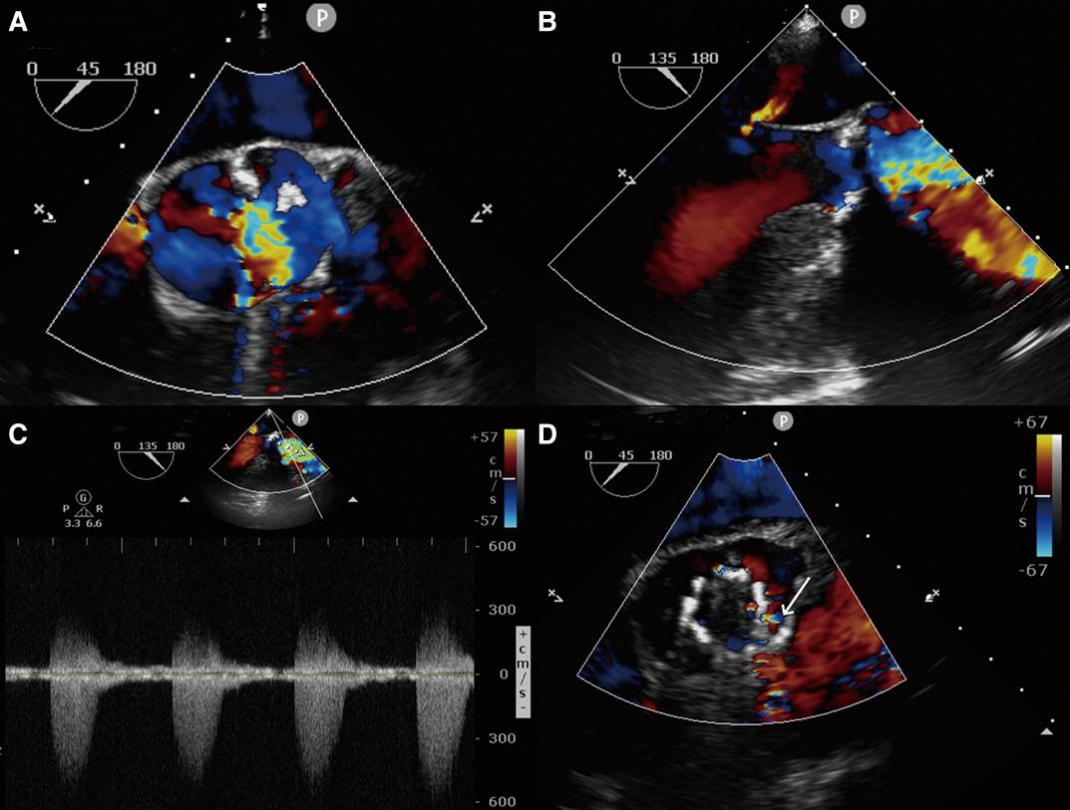
Pre-operative and post-operative echocardiography. (*A–C*) Pre-operative echocardiography: severe aortic stenosis; (*D*): Post-operative echocardiography: slight paravalvular leakage (white arrow).

**Figure 3:**
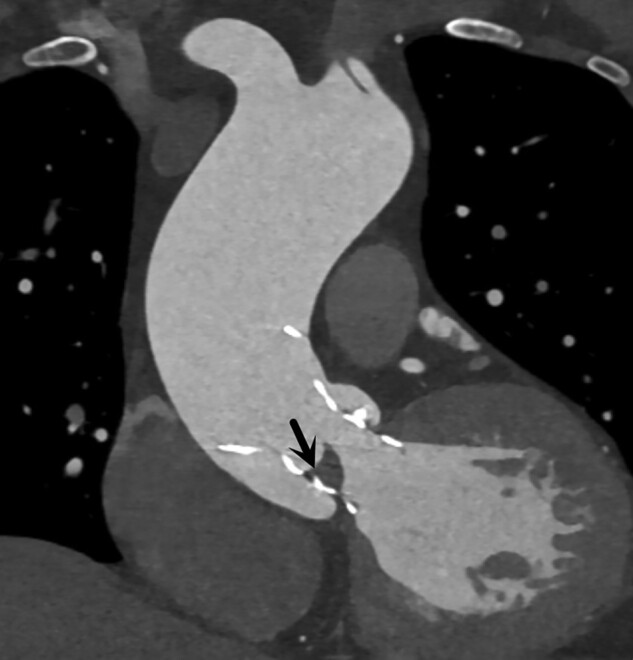
Computer tomography on Day 30. Hypo-attenuated leaflet thickening in the right leaflet (black arrow).

When the symptoms of Sjögren's syndrome were relieved, the patient was referred to structural cardiologists. Physical examination: blood pressure 120/79 mmHg, heart rate 85 beats/min, and cardiac murmur remained unchanged. On repeat transthoracic echocardiography, severe AS and mild TR were confirmed. The main cardiac parameters included peak velocity of 5.53 m/s, mean gradient of 74 mmHg, orifice area of 0.68 cm^2^, left ventricular diameters of 49/29 mm (diastole/systole), and ejection fraction of 0.71. Aortic computed tomography angiography (CTA) showed a bicuspid aortic valve (BAV) of Type 0 and provided information regarding the aortic valve annulus and left ventricular outflow tract (*Figure 1*). Combined with relevant clinical examinations (*Table 1*), the patient was diagnosed with severe AS and Sjögren's syndrome, combined with leukopaenia (1.26 × 10^9^/L, diagnosed according to criteria of <4.0 × 10^9^/L) and thrombocytopaenia (35 × 10^9^/L, diagnosed according to criteria of <4.0 × 10^9^/L).^4^ After multidisciplinary consultations and communication with family members, a TaurusElite 26 mm valve (Suzhou Peijia Medical Co., Ltd, China) was implanted by transcatheter aortic valve replacement (TAVR) on Day 7 of admission, following 3D printing simulation and evaluation. Intraoperative aortography and transoesophageal echocardiography indicated successful implantation of the prosthesis with minor perivalvular leakage and no coronary artery obstruction (*Figure 2*, Supplementary material online, *Videos S1–S3*). Invasive gradients showed the peak systolic gradient dropped from 135 mmHg preoperatively to 0 mmHg. Methylprednisolone 28 mg/day and hydroxychloroquine sulfate 400 mg/day were given to achieve systemic immunomodulation/immunosuppression, clopidogrel 75 mg/day to antithrombosis and sacubitril/valsartan 100 mg/day was continued. Transthoracic echocardiography before discharge showed a peak velocity of 1.41 m/s, mean gradient of 5 mmHg and orifice area of 1.6 cm^2^. Left ventricular diameters were 46/27 mm (diastole/systole) and ejection fraction was 0.72. On Day 44 post-TAVR, the symptoms of AS resolved and no obvious murmur was heard in the aortic valve auscultation area. Routine post-TAVR CTA showed a hypo-attenuated leaflet thickening (HALT) (*Figure 3*), and warfarin was given to replace clopidogrel with dose titration to maintain a target international normalized ratio of 1.5–2.5.^5^

**Table 1:** Laboratory findings on admission

	Reference range	On arrival	In cardiac catheterization	1 day after TAVR	Pre-discharge
WBC (10^9^/L)	3.5–9.5	1.26	4.75	6.13	6.06
PLT (10^9^/L)	125–350	35	78	30	48
Albumin (g/L)	40–55	33.5	36.5	31.6	
ATA III (%)	75–114	65.1			97.7
INR	0.7–1.3	1.01	0.9	0.91	
NT-proBNP (pg/mL)	<450	2240	3030	4530	
ACT (s)	100–155		163		
ANA	<1:40	>1:2560			
SSA antibody/RO-60		Positive			
SSB antibody/RO-52		Positive			
CENP-B antibody		Positive			
C3 complement (g/L)	0.9–1.8	0.659			0.914
C4 complement (g/L)	0.1–0.4	0.069			0.165
Saliva flow rate (mL/15 min)	>1.5	0.5			
Tear film breakup time (mean) (s)	>7	6.4 L/0.6 R			
ACA (Ig G)	<8	7.98			
ACA (Ig M)	<8	2.73			
anti-β2GPI	<16	6.23			

WBC, white blood cell; PLT, platelet count; INR, international normalized ratio; ATA, anti-thrombin antibody; NT-proBNP, N-terminal pro-B-type natriuretic peptide; ACT, activated coagulation time; ANA anti-nuclear antibody; SSA, Sjögren's syndrome A; SSB, Sjögren's syndrome B; CENP-B, centromere protein B; L, left-sightedness; R, right-sightedness; ACA, anticardiolipin antibody; anti-β2GPI, anti-β2-glycoprotein-I.

## Discussion

To our knowledge, this is the first report on the decision-making of TAVR for severe AS in a patient with Sjögren's syndrome and severe thrombocytopaenia. For young patients, we prefer to perform surgical aortic valve replacement (SAVR) according to ESC/EACTS valve management guidelines. However, this patient with thrombocytopaenia cannot tolerate cardiopulmonary bypass, and the larger surgical trauma would delay the patient's recovery. After a comprehensive evaluation, structural cardiologists and cardiac surgeons unanimously agreed that the hazards of SAVR surpassed the potential benefits and TAVR was the best choice for her at that time. If the patient refused, we would follow the valve management guidelines for conservative treatment, and pay attention to thrombocytopaenia and Sjögren's syndrome. This case highlights several noteworthy considerations. Firstly, the co-existing risks of bleeding and thrombosis (Padua Thrombosis Score 7, PREDICT-TAVR Score 11) increased the complexity and difficulty of proper antithrombotic therapy. Thrombocytopaenia would result in a high bleeding risk that might be aggravated by the use of anticoagulants in TAVR.^5^ To reduce bleeding risk, we used 3D printing to determine the depth of the implant and simulate release. This can assist operators in accurate positioning during intervention, effectively reducing the frequency of pre-/post-balloon expansion and valve-crossing procedures, thereby shortening the surgical duration. Additionally, the history of cerebral infarction and the process of implantation may promote the activation of coagulation factors. Immune complex aggregation caused by Sjögren's syndrome could also lead to valve thrombosis. For all we know, the optimal antithrombotic strategy for patients with both bleeding and thrombosis risks was not clear yet. Therefore, we intend to refer to the strategy of patients with thrombocytopaenia after percutaneous coronary intervention, i.e. discontinuing clopidogrel when platelet count (PLT) ≤ 50 × 10^9^/L. Forty-four-day post-TAVR CTA showed HALT, and clopidogrel was switched to warfarin, considering the high risk of thrombosis associated with Sjögren's syndrome.

Secondly, patients with leukopaenia and long-term use of immunosuppressants have a high risk of perioperative infection.^6^ The decreased salivary secretion of patients with Sjögren's syndrome would lead to an increased risk of oral infection and aspiration pneumonia due to tracheal intubation. Subsequently, increasing the risk of infective endocarditis. We prophylactically used cefazolin sodium for anti-infection after TAVR. The immunosuppressants were suspended to prevent perioperative infection, and low-dose immunoglobulin was infused to improve immunity function without increasing the risk of stroke.

Third, lifelong management of implanted valves is also a common concern. Ahmad *et al.* reported a 75-year-old female patient who developed structural valve deterioration due to calcification and secondary cryoglobulinaemic vasculitis by Sjögren's syndrome 4 years after TAVR; thus, the possibility of bioprosthetic valve dysfunction and subsequent intervention should be considered before the operation.^7^ We used a pre-expanded balloon to observe the coronary perfusion and a 3D printing model was applied to determine the optimal size of the prosthetic valve to obtain a larger effective orifice area.^8^ Eventually, the prosthetic valve achieved satisfying function without serious deformation, potentially contributing to an extended valve lifespan. Post-operative hydroxychloroquine sulfate was used to reduce valve damage from inflammation, following consultation with rheumatologists.

Patients with BAV often have an elliptical annulus, asymmetric calcification, unequal leaflet size, and concomitant widening of the ascending aorta.^9^ These unfavourable anatomical characteristics increase the likelihood of inappropriate implant depth and incomplete expansion. Meanwhile, low calcification provides insufficient anchoring force, making valve migration likely. These may promote the occurrence of HALT or even surgical failure. We believe that 3D printing technology can help determine the optimal oversizing to obtain sufficient anchoring force and post-balloon expansion can be employed to ensure complete expansion of the prosthesis.

There were also some pitfalls in the treatment of this case. The PLT of this patient decreased from 78 × 10^9^/L preoperatively to 33 × 10^9^/L postoperatively, we suspected that heparin-induced thrombocytopaenia (HIT) occurred. However, HIT in patients with Sjögren's syndrome has not been reported in the previous literature, leading to a lack of reference for formulating treatment plans. A review by Chalayer *et al.* found that fragment crystallisable (Fc) receptors have similar expression in Sjögren's syndrome and HIT, suggesting that patients with Sjögren's syndrome may have a higher risk of HIT.^10^ Therefore, we considered that bivalirudin might have been more appropriate than heparin in TAVR procedures for patients with Sjögren's syndrome. Although further research is needed to confirm the mechanism and epidemiology of HIT in patients with Sjögren's syndrome, low molecular weight heparin or bivalirudin may be the *modus vivendi* to reduce the risk.

## Conclusion

The mechanism of the damage of Sjögren's syndrome to the native valve and the prosthetic valve was still unclear, but we should fully consider the effect of the autoimmune mechanism on bleeding/thrombosis risk, post-operative infection risk and prosthetic valve life. The current report may serve as a reference for TAVR and perioperative management in these patients. The patient will undergo close follow-up for monitoring of valve function, coagulation and immune system with adjustments in treatment as required. We hope the follow-up work could provide a scientific basis for studying the impact of Sjögren's syndrome on cardiac valves and lifelong management of valves.

## Supplementary Material

ytad622_Supplementary_Data

## Data Availability

The data underlying this article are available in the article and in its online supplementary material.

## Lead author biography

Yan Li is the director of the Department of Cardiology, Tangdu Hospital, Air Force Medical University. She is a fellow of the European Society of Cardiology, global ADR technology instructor and left atrial appendage occlusion instructor. She has been engaged in the treatment of structural heart disease and complex coronary heart disease for a long time. Yan Li is the director of the Department of Cardiology, Tangdu Hospital, Air Force Medical University. She is an expert in heart failure and structural heart disease.
