# Supplementary material for: A case report of transcatheter aortic valve replacement in a patient with Sjögren's syndrome and aortic stenosis
Source: Eur Heart J Case Rep. 2023 Dec 11;8(1):ytad622. doi: 10.1093/ehjcr/ytad622 (PMC10751622; doi:10.1093/ehjcr/ytad622)
Supplement: ytad622_Supplementary_Data [file ytad622_supplementary_data.zip › supplyment.doc]

**Video 1. Severe aortic stenosis in preoperative echocardiography.** In both the short and long axis, aortic valve cannot be fully opened, with high blood flow velocity.

**Video 2. Cardiography in operation.** The prosthesis was fully unfolded without significant deformation; no blockage in the coronary artery.

**Video 3. Paravalvular leakage** **after implantation .** In the short axis, small light spots appeared on the outer side of the prosthesis without significant central reflux beam.
